# Effects of the prenatal and postnatal nurturing environment on the phenotype and gut microbiota of mice with polycystic ovary syndrome induced by prenatal androgen exposure: a cross-fostering study

**DOI:** 10.3389/fcell.2024.1365624

**Published:** 2024-03-25

**Authors:** Akari Kusamoto, Miyuki Harada, Ayaka Minemura, Asami Matsumoto, Kentaro Oka, Motomichi Takahashi, Nanoka Sakaguchi, Jerilee M. K. Azhary, Hiroshi Koike, Zixin Xu, Tsurugi Tanaka, Yoko Urata, Chisato Kunitomi, Nozomi Takahashi, Osamu Wada-Hiraike, Yasushi Hirota, Yutaka Osuga

**Affiliations:** ^1^ Department of Obstetrics and Gynecology, Faculty of Medicine, University of Tokyo, Tokyo, Japan; ^2^ R&D Division, Miyarisan Pharmaceutical Co., Ltd., Saitama, Japan; ^3^ Department of Obstetrics and Gynecology, Faculty of Medicine, University of Malaya, Kuala Lumpur, Malaysia

**Keywords:** androgen, cross-fostering model, delayed effect of prenatal exposure, gastrointestinal microbiome, polycystic ovary syndrome (PCOS)

## Abstract

The gut microbiome is implicated in the pathogenesis of polycystic ovary syndrome (PCOS), and prenatal androgen exposure is involved in the development of PCOS in later life. Our previous study of a mouse model of PCOS induced by prenatal dihydrotestosterone (DHT) exposure showed that the reproductive phenotype of PCOS appears from puberty, followed by the appearance of the metabolic phenotype after young adulthood, while changes in the gut microbiota was already apparent before puberty. To determine whether the prenatal or postnatal nurturing environment primarily contributes to these changes that characterize prenatally androgenized (PNA) offspring, we used a cross-fostering model to evaluate the effects of changes in the postnatal early-life environment of PNA offspring on the development of PCOS-like phenotypes and alterations in the gut microbiota in later life. Female PNA offspring fostered by normal dams (exposed to an abnormal prenatal environment only, fostered PNA) exhibited less marked PCOS-like phenotypes than PNA offspring, especially with respect to the metabolic phenotype. The gut microbiota of the fostered PNA offspring was similar to that of controls before adolescence, but differences between the fostered PNA and control groups became apparent after young adulthood. In conclusion, both prenatal androgen exposure and the postnatal early-life environment created by the DHT injection of mothers contribute to the development of PCOS-like phenotypes and the alterations in the gut microbiota that characterize PNA offspring. Thus, both the pre- and postnatal environments represent targets for the prevention of PCOS and the associated alteration in the gut microbiota in later life.

## 1 Introduction

Polycystic ovary syndrome (PCOS) is the most common endocrine disorder of reproductive-age women, affecting up to 15% of this group (Escobar-Morreale, 2018). PCOS is diagnosed using the Rotterdam criteria, the most widely used criteria for PCOS, which require two of the following to be present: i) hyperandrogenism, ii) ovulatory dysfunction, or iii) polycystic ovarian morphology (Fauser et al., 2004). Although the pathophysiology of PCOS has not been fully elucidated, owing to its heterogeneity and complexity, hyperandrogenism, ovulatory dysfunction, abnormal gonadotropin secretion, and insulin resistance have been implicated (Dumesic et al., 2015b; Harada, 2022); and these reproductive and metabolic abnormalities interact and exacerbate one another.

It has been reported that the daughters of women with PCOS are at a high risk of developing PCOS (Filippou and Homburg, 2017; Crisosto et al., 2019; Risal et al., 2019). PCOS is considered as multifactorial disorder with polygenetic and environmental contributions (Dunaif, 2016; Escobar-Morreale, 2018). Out of these factors, prenatal androgen exposure has been recognized as a critical environmental contributor to the development of PCOS. It has been shown that the daughters of women with PCOS are prenatally exposed to high concentrations of androgens during fetal life (Sir-Petermann et al., 2002; Maliqueo et al., 2013). In addition, daughters born to women with PCOS have a longer anogenital distance (AGD), the distance from the center of the anus to the posterior fourchette, which serves as a biomarker of intrauterine exposure to excess androgens (Barrett et al., 2018; Perlman et al., 2020). Furthermore, numerous studies have reported that prenatally androgenized (PNA) animals, including rodents, sheep, and rhesus monkeys, exhibit PCOS-like reproductive and metabolic phenotypes in adulthood (Dumesic et al., 2015a; Risal et al., 2019). However, the mechanism by which prenatal androgen exposure is involved in the etiology of PCOS in later life is unknown.

It was recently reported that the gut microbiome of adult women with PCOS differs from that of healthy individuals (Lindheim et al., 2017; Insenser et al., 2018; Torres et al., 2018). In addition, rodents with PCOS induced by the long-term administration of androgens or aromatase inhibitors during puberty or adulthood have a different gut microbiome to controls in adulthood (Kelley et al., 2016; Arroyo et al., 2019; Torres et al., 2019a; Zheng et al., 2020). Furthermore, the transplantation of feces from or co-housing with healthy controls ameliorates the PCOS-like phenotype of such rodents, suggesting that changes in the gut microbiota are not merely a result of the pathology characterizing PCOS, which has both reproductive and metabolic components, but that the gut microbiota may play an etiologic role in PCOS (Guo et al., 2016; Torres et al., 2019b).

Based on these findings, we hypothesized that prenatal androgen exposure would induce dysbiosis of the gut microbiota early stage of life and that this would lead to the development of PCOS in later life. In the previous our study, we characterized the temporal relationship between alterations in the gut microbiota and the development of PCOS-like phenotypes between weaning and adulthood using prenatally androgenized female mice (Kusamoto et al., 2021). We found that abnormalities in the gut microbiota appear as early as or even before PCOS-like phenotypes manifest in PNA offspring, which suggests that the gut microbiota in early life represents a potential target for the prevention of PCOS in later life. However, the findings of the study raised a question: which of the prenatal environment or the postnatal nurturing environment contributes more to the etiology of PCOS phenotypes and the alterations in the gut microbiota, or are both important? To answer this question, in the present study, we used a cross-fostering model. The PCOS-like phenotypes and the differences in the gut microbiota in PNA offspring fostered with a control mother (fostered PNA) and PNA offspring kept with their own mothers were compared to control. To the best of our knowledge, this is the first study to separately demonstrate the effects of the prenatal and postnatal early-life environments on the development of PCOS phenotypes and the gut microbiota in later life.

## 2 Materials and methods

### 2.1 PNA model and cross-fostering design

**FIGURE 1:**
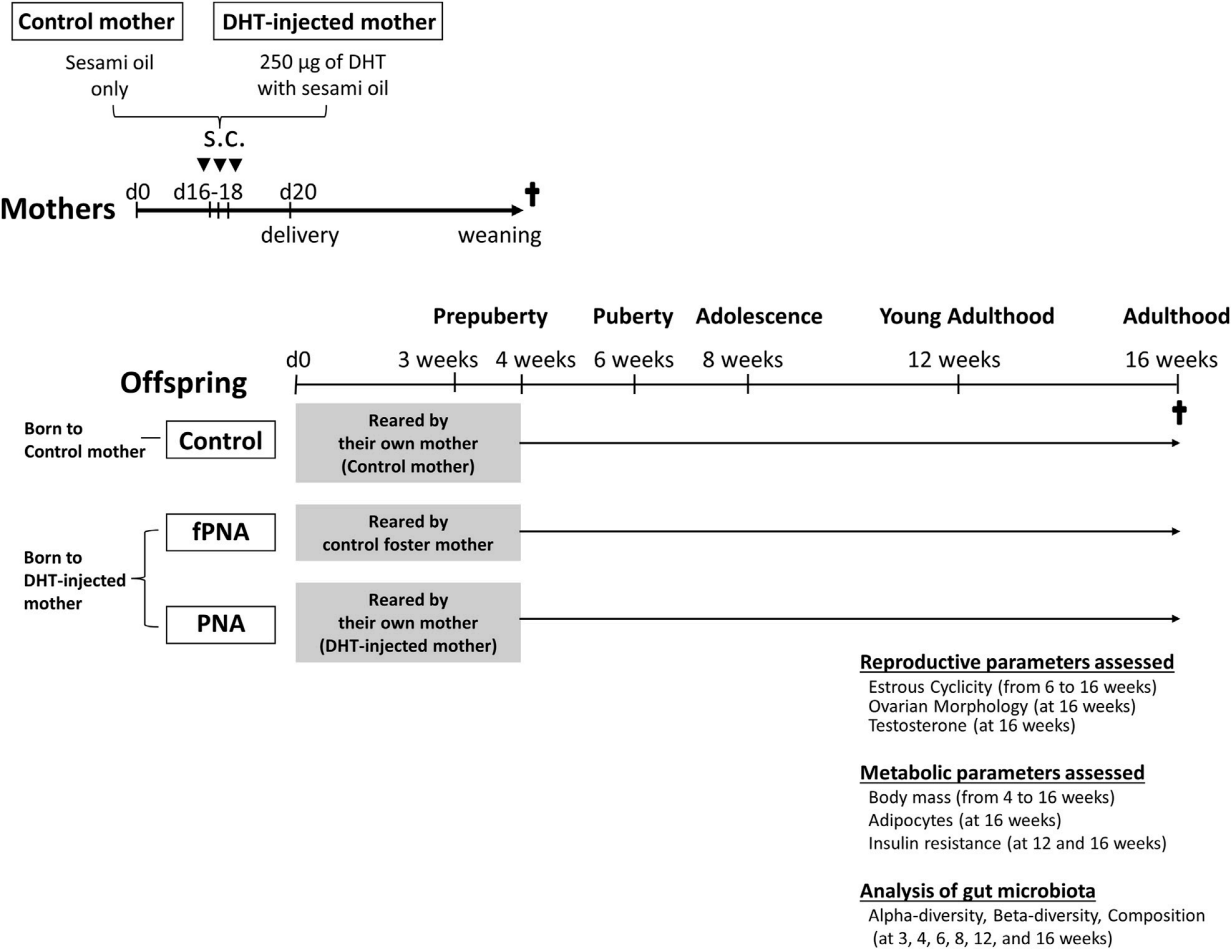
Experimental design. Scheme showing how prenatally androgenized (PNA) offspring that were cross-fostered were generated and how the outcomes were evaluated. Pregnant dams were subcutaneously (s.c.) injected on days 16, 17, and 18 of gestation with either 0.1 mL sesami oil (control mothers) or 0.1 mL sesami oil containing 250 μg of dihydrotestosterone (DHT-injected mothers). The offspring of control mothers were reared by their own mothers for 4 weeks, and female pups born to DHT-injected mothers were reared by their own mothers (PNA group) or control foster mothers (fostered PNA group) for 4 weeks. The development of a PCOS-like reproductive phenotype, characterized by abnormal estrous cyclicity (*n* = 23 to 25 per group), ovarian histology (*n* = 10 to 12 per group), and serum testosterone concentration (*n* = 21 to 23 per group); and the development of a metabolic phenotype, including abnormal body mass (*n* = 23 to 25 per group), size of visceral adipocytes (*n* = 11 to 14 per group), and results of insulin tolerance testing (ITT) (*n* = 17 to 20 per group), were assessed at each time point. The gut microbiota of the female offspring was analyzed at various time points (*n* = 3 to 5 per group), as shown.

Cross-fostering (Mccormack et al., 2022) was used in a mouse model of PCOS induced by prenatal androgen exposure to evaluate the effects of the prenatal and postnatal early-life environments on PCOS-like phenotypes and the gut microbiota. The PNA model was generated by the injection of pregnant mice with DHT, as previously reported (Sullivan and Moenter, 2004; Moore et al., 2013; Caldwell et al., 2014; Risal et al., 2019; Kusamoto et al., 2021; Wu et al., 2021). Eight-week-old male and female C57BL/6 mice, aged 8–12 weeks, were provided by Japan SLC Inc (Hamamatsu, Japan). The mice were maintained under specific pathogen-free conditions and under a 12-h light/dark cycle, with *ad libitum* access to water and food. After 1 week of adaptation to their surroundings, the female mice were paired with male mice and subsequently checked for the presence of copulatory plugs. The date of identification of a plug was regarded as day 1 of gestation. The experimental design is shown in Figure 1. Pregnant dams were subcutaneously injected on days 16, 17, and 18 of gestation with either 0.1 mL sesami oil (control mothers) or 0.1 mL sesami oil containing 250 μg of DHT (Sigma-Aldrich, St. Louis, MO, United States; DHT-injected mothers). Within 12 h of birth, the pups were exchanged between mothers who gave birth at the same time. The offspring of control mothers were all reared by their own mothers for 4 weeks, and offspring born from DHT-injected mothers were reared by their own mothers (PNA group) or control foster mothers (fostered PNA group) for 4 weeks. There were no differences in maternal nurturing behavior between these groups, including breastfeeding. All the female pups were weaned at 4 weeks of age and subsequently housed separately from their dams. The three groups of offspring were kept separately, with three or four pups per cage. The PCOS-like phenotypes and gut microbiota of the female offspring in control, fostered PNA, and PNA groups were then analyzed at various time points.

The AGD of the mice was measured early in their lives to confirm the androgenization of the female offspring. PNA and fostered PNA offspring had longer AGDs than control offspring (PNA, 4.53 ± 0.72 mm; fostered PNA, 4.82 ± 0.49 mm; control, 3.53 ± 0.47 mm (*p* < 0.001 *vs*. the control group) at 4 weeks; and PNA, 5.98 ± 0.67 mm; fostered PNA, 6.00 ± 0.51 mm; control, 4.05 ± 0.27 mm (*p* < 0.001 *vs*. the control group) at 6 weeks, confirming that the PNA and fostered PNA offspring had been exposed to the androgen *in utero*. The mice were euthanized under isoflurane anesthesia, and blood, ovarian, parametrial fat, and fecal samples were collected. The PCOS-like reproductive phenotype was confirmed by assessing estrous cyclicity, ovarian histology, and serum testosterone concentration; and the metabolic phenotype was identified by assessing body mass, the size of the visceral adipocytes, and insulin tolerance testing (ITT), using the methods described in our previous publication (Kusamoto et al., 2021).

To evaluate the effects of the prenatal and early-life environments on the development of the gut microbiota of female offspring, next-generation sequencing (NGS) and bioinformatic analysis of the 16S rRNA genes were performed using DNA extracted from fecal samples from the three groups that were obtained at 3, 4, 6, 8, 12, and 16 weeks of age. These ages correspond to prepuberty (3 and 4 weeks), puberty, adolescence, young adulthood, and adulthood, respectively. In addition, the gut microbiota of control and DHT-injected mothers were analyzed after 3 days of injections, to determine whether the gut microbiota of the DHT-treated mothers had been altered. All the procedures used in the study were performed in compliance with the guidelines and regulations of the University of Tokyo Committee on the Use and Care of Animals, and the study was approved by the committee (approval number: P21-005).

### 2.2 Estrous cyclicity

Estrous cyclicity was analyzed using well-established methods (Moore et al., 2013; Wang et al., 2018). Vaginal cells were collected from female offspring every morning from 6 to 16 weeks of age, and the stages of the estrous cycle recognized by light microscopic examination were diestrus, proestrus, estrus, and metestrus, as previously described (Cora et al., 2015; Ajayi and Akhigbe, 2020). Each stage of the estrous cycle was defined by assessing the proportions of neutrophils, nucleated epithelial cells, and anuclear keratinized epithelial cells. Briefly, in diestrus, neutrophils are predominately present; in metestrus, neutrophils and anuclear keratinized epithelial cells are present; in estrus, anuclear keratinized epithelial cells are characterized; and in proestrus, small, round, nucleated epithelial cells are visible.

### 2.3 Ovarian and adipose histology

The ovaries and parametrial fat samples were collected from control, fostered PNA, and PNA mice at 16 weeks of age. The ovaries and adipose tissue were fixed, embedded in paraffin, and 5-μm thick sections were prepared. These sections were stained with hematoxylin and eosin. The ovarian sections were examined by two investigators using an Olympus BX50 Fluorescence Microscope (Olympus, Tokyo, Japan). The number of atretic antral follicles were counted across the entire ovary, as previously described (Kusamoto et al., 2021). Atretic follicles were recognized by the presence of an attenuated granulosa cell layer, shrinkage, and degenerate oocyte nuclei (Osman, 1985). For the adipose tissue samples, two representative micrographs were obtained per sample at ×40 magnification using a light microscope (Olympus BX50 Fluorescence Microscope). The sizes of the adipocytes were quantified using ImageJ software (National Institutes of Health, Bethesda, MD, United States), as previously described (Benrick et al., 2017; Kusamoto et al., 2021).

### 2.4 Serum testosterone concentration

To measure the serum testosterone concentration, blood samples were collected from the mice at 16 weeks of age by cardiac exsanguination under isoflurane anesthesia at 09:00, because it exhibits diurnal variation. The samples were centrifuged and the separated serum was stored at −80°C until assayed. The serum concentration of testosterone was measured using an ELISA kit for testosterone (RRID: AB_2848196; Enzo Life Sciences, Farmingdale, NY, United States).

### 2.5 Insulin tolerance testing (ITT)

To evaluate insulin resistance, ITT was performed, as previous described (Ayala et al., 2010). Briefly, mice were intraperitoneally administered insulin (0.75 IU/kg body mass) after fasting for 5 h, then the glucose concentrations of blood samples obtained from a tail vein were measured using a glucometer (LAB Gluco, ForaCare Japan, Tokyo, Japan) 0, 15, 30, 60, and 120 min later.

### 2.6 DNA extraction and NGS of the fecal bacterial 16S rRNA genes

DNA extraction was performed as described previously (Kobayashi et al., 2013), with slight modifications. Briefly, fecal samples stored at −80°C were suspended in 600 μL of TE buffer and glass beads were added to the samples. The samples were then homogenized at 7,000 rpm for 20 s using a MagNA Lyser (Roche Diagnostics GmbH, Mannheim, Germany). Following the addition of 100 µL of 10% sodium dodecyl sulfate (Fujifilm Wako Pure Chemical Co., Inc., Osaka, Japan) and 600 µL of Phenol/Chloroform/Isoamyl alcohol solution (Nippon Gene Co., Ltd., Tokyo, Japan), the samples were homogenized in the same way. The samples were then incubated at 70°C for 10 min and homogenized similarly again. The samples were then centrifuged at 20,400 × *g* for 5 min and 600 µL of each supernatant was transferred to a new 1.5 mL tube and mixed with 600 µL of 2-propanol (Kanto Chemical Co., Inc., Tokyo, Japan) and 60 µL of 3 M sodium acetate (Nippon Gene). After gentle shaking, the samples were centrifuged at 20,400 × *g* for 5 min and the supernatants were discarded. The DNA pellets were washed twice with 70% ethanol (Kanto Chemical), dried with a centrifugal evaporator (Tomy Seiko Co., Ltd., Tokyo, Japan), and dissolved in 200 µL of TE buffer. One microliter of RNase (10 mg/mL; Nippon Gene) was added to each sample, and they were incubated at 37°C for 1 h. Finally, the extracted DNA was purified using a High Pure PCR Template Preparation Kit (Roche Diagnostics), according to the manufacturer’s instructions. The variable V3-4 regions of the 16S rRNA gene were amplified by PCR using the TaKaRa Ex Taq^®^ Hot Start Version (Takara Bio Inc., Shiga, Japan) and the universal 16S primer sets 341F and 805R, which contain the Illumina index and sequencing adapter overhangs. The amplicons were purified using SPRIselect (Beckman Coulter, Inc., Brea CA, United States) and the DNA was quantified using a QuantiFluor One dsDNA system (Promega Corporation). Mixed samples were prepared by pooling approximately equal amounts of each amplified DNA sample and sequenced using a MiSeq Reagent Kit v3 (600 cycles) and MiSeq sequencer (Illumina, Inc., San Diego, CA, United States), according to the manufacturer’s instructions.

### 2.7 Bioinformatic analysis of the fecal bacterial 16S rRNA genes

The 16S rRNA gene sequence data generated by the MiSeq sequencer were processed using the quantitative insights into microbial ecology 2 (QIIME 2 2021.2) pipeline (Bolyen et al., 2017). Raw sequence data were demultiplexed and quality-filtered using the q2-demux plugin, then denoised using DADA2 (Ombrello, 2020). All the amplicon sequence variants (ASVs) were aligned using MAFFT (Katoh et al., 2002), and their phylogeny was assessed using FastTree2 (Price et al., 2010). Taxa were assigned to the ASVs using the q2-feature-classifier (Bokulich et al., 2018) classify-sklearn naïve Bayes taxonomy classifier against SILVA 138.1 (Pruesse et al., 2007).

For diversity analysis, the number of sequence reads was set to the minimum reads among samples using the qiime diversity core-metrics-phylogenetic method (Bolyen et al., 2017). The within-community diversity (α-diversity) was calculated using the Chao1 index. To compare the microbial composition of the samples, β-diversity was used, and this was calculated using the unweighted UniFrac distances. PCoA was used to generate two-dimensional plots from the resulting distance matrices. LEfSe with default settings was used to identify the bacterial taxa that significantly differed in abundance between the groups (Segata et al., 2011).

### 2.8 Statistical analysis

One-way ANOVA, followed by post-hoc comparisons using the Tukey-Kramer honest significant difference test, was used to compare the features of the PCOS phenotypes between groups at the same time points, using JMP Pro 17 software (SAS Institute Inc, Cary, NC, United States). *p* < 0.05 was considered to represent statistical significance. The Mann-Whitney U test was used to compare the Chao1 index values. The difference of each group in the community structure was compared using permutational multivariate analysis of variance (PERMANOVA).

## 3 Results

### 3.1 Female offspring exposed to androgens prenatally exhibit disrupted estrous cycles during all stages of growth, regardless of their postnatal early-life environment

**FIGURE 2:**
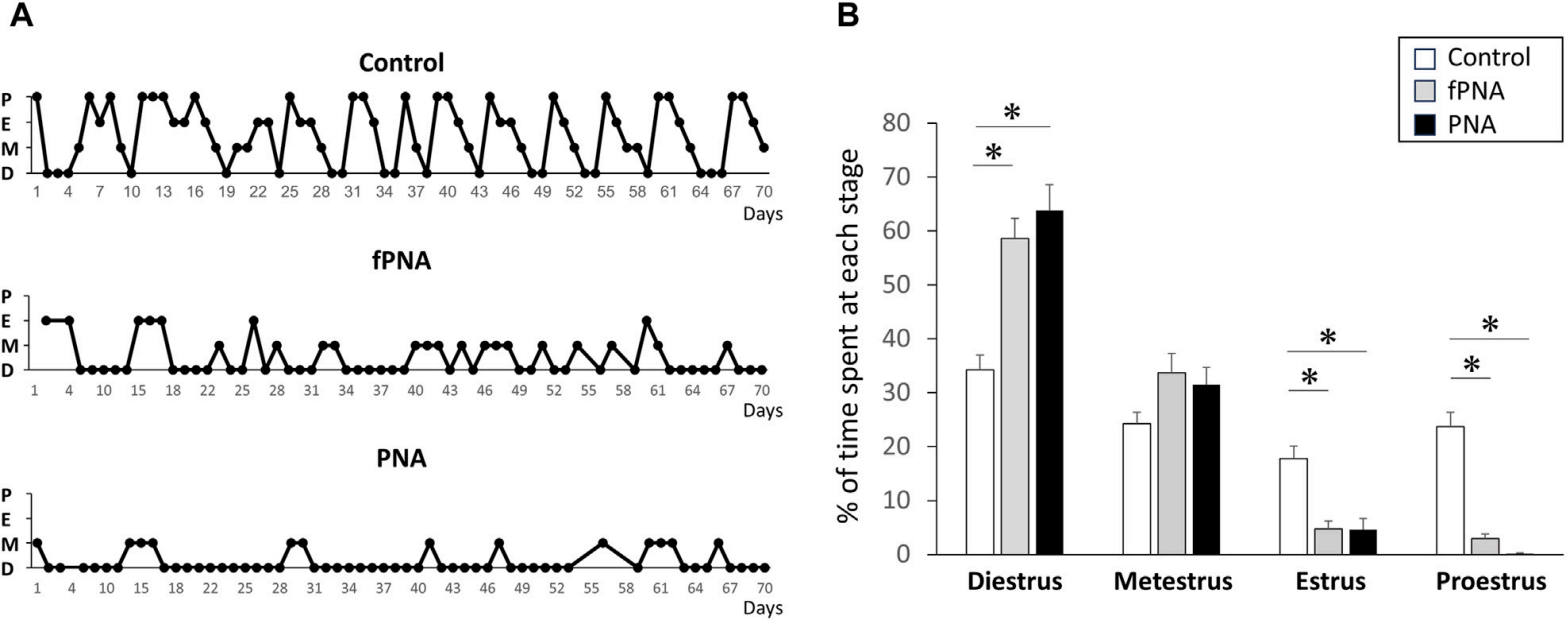
Estrous cyclicity. PNA and fostered PNA offspring exhibited disruption of their estrous cycles at all the time points, while control offspring exhibited normal cycles. **(A)** Representative estrous cycles for control, fostered PNA, and PNA offspring are shown. **(B)** The percentage of time spent at each estrous stage. PNA and fostered PNA offspring showed increased percentage of days in the estrous cycle spent in diestrus and decreased percentage of days spent in proestrus and estrus. fPNA, fostered PNA. D, diestrus; M, metestrus; E, estrus; P, proestrus.

PNA and fostered PNA offspring exhibited disrupted estrous cycles during all stages of their life, while control offspring exhibited normal cycles. Compared to control, PNA and fostered PNA offspring represented increased percentage of days in the estrous cycle spent in diestrus and decreased percentage of days spent in proestrus and estrus. Therefore, the disruption to the estrous cycle induced by prenatal androgen exposure is not normalized by changing the postnatal early-life environment (Figure 2).

### 3.2 Prenatal androgen exposure induces changes in ovarian morphology and increases the serum testosterone concentration, and this change in testosterone concentration is affected by the postnatal early-life environment

**FIGURE 3:**
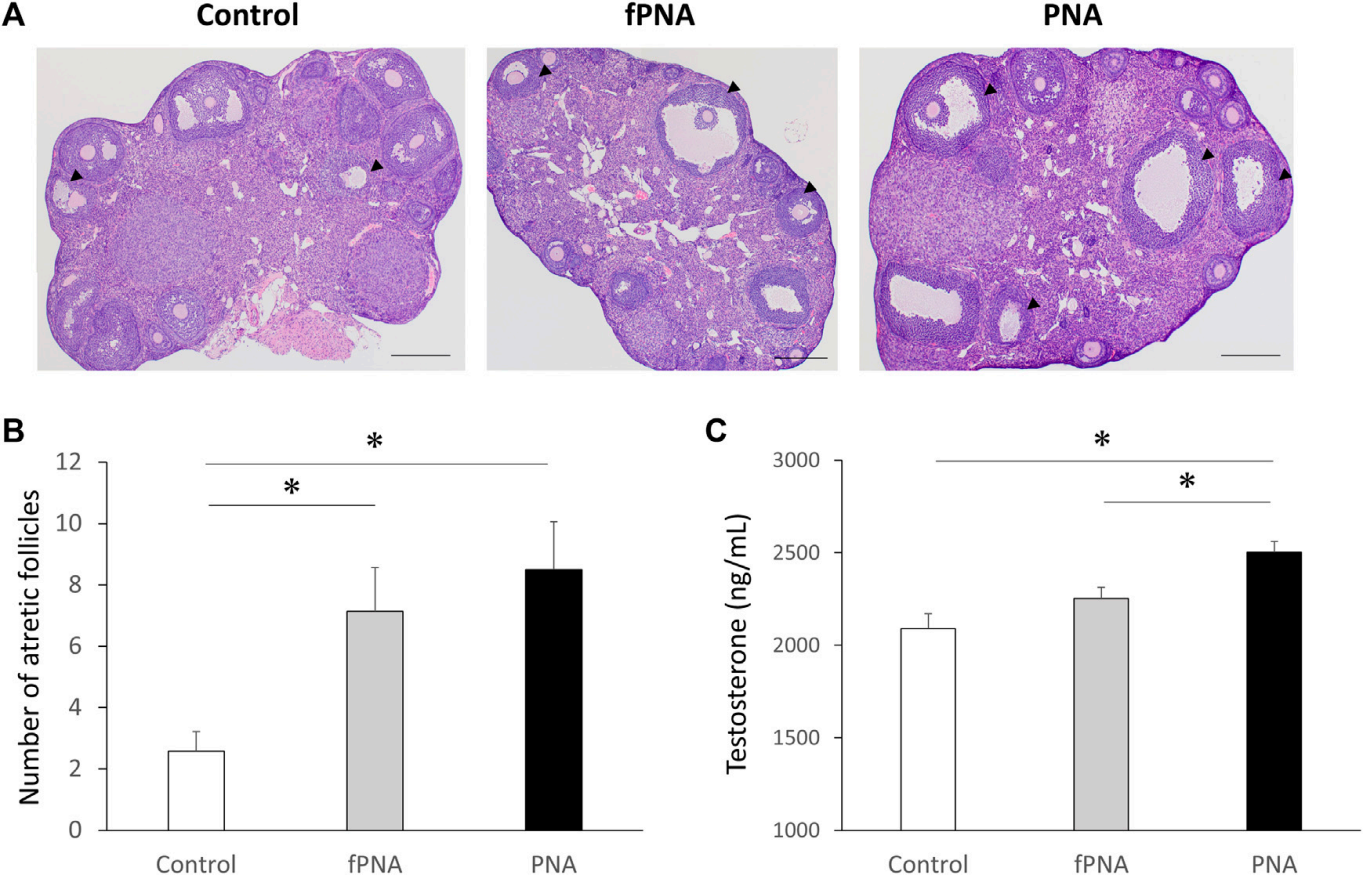
Ovarian morphology and serum testosterone concentrations. **(A)** Representative sections through an ovary, **(B)** the number of atretic antral follicles, and **(C)** the serum testosterone concentration in mice of the control, fostered PNA, and PNA groups at 16 weeks of age. Cystic follicles with large antra and degenerate granulosa cells are apparent in the ovaries of the fostered PNA and PNA offspring. There were larger numbers of atretic follicles in both fostered PNA and PNA offspring than in control offspring. The mean serum testosterone concentration of PNA offspring was significantly higher than that of control and fostered PNA offspring. Scale bars: 400 μm. Values are means ± SEMs. *p < 0.05 according to one-way-ANOVA, followed by *post-hoc* Tukey-Kramer honest significant difference test analysis. fPNA, fostered PNA.

Figure 3A shows representative histological images of ovaries from control, fostered PNA, and PNA offspring. Follicle counting revealed a larger number of atretic antral follicles in both the fostered PNA and PNA offspring than in control offspring (*p* = 0.011, *p* = 0.042, respectively) at the age of 16 weeks (Figure 3B) and there was no difference between the fostered PNA and PNA offspring. As shown in Figure 3C, the PNA offspring had significantly higher testosterone concentrations than the control (*p* < 0.001) and fostered PNA groups (*p* = 0.039) at 16 weeks of age, but there was no difference between fostered PNA and control offspring.

### 3.3 The prenatal androgen-induced increase in body mass and hypertrophy of parametrial adipocytes are attenuated by changing the postnatal early-life environment

**FIGURE 4:**
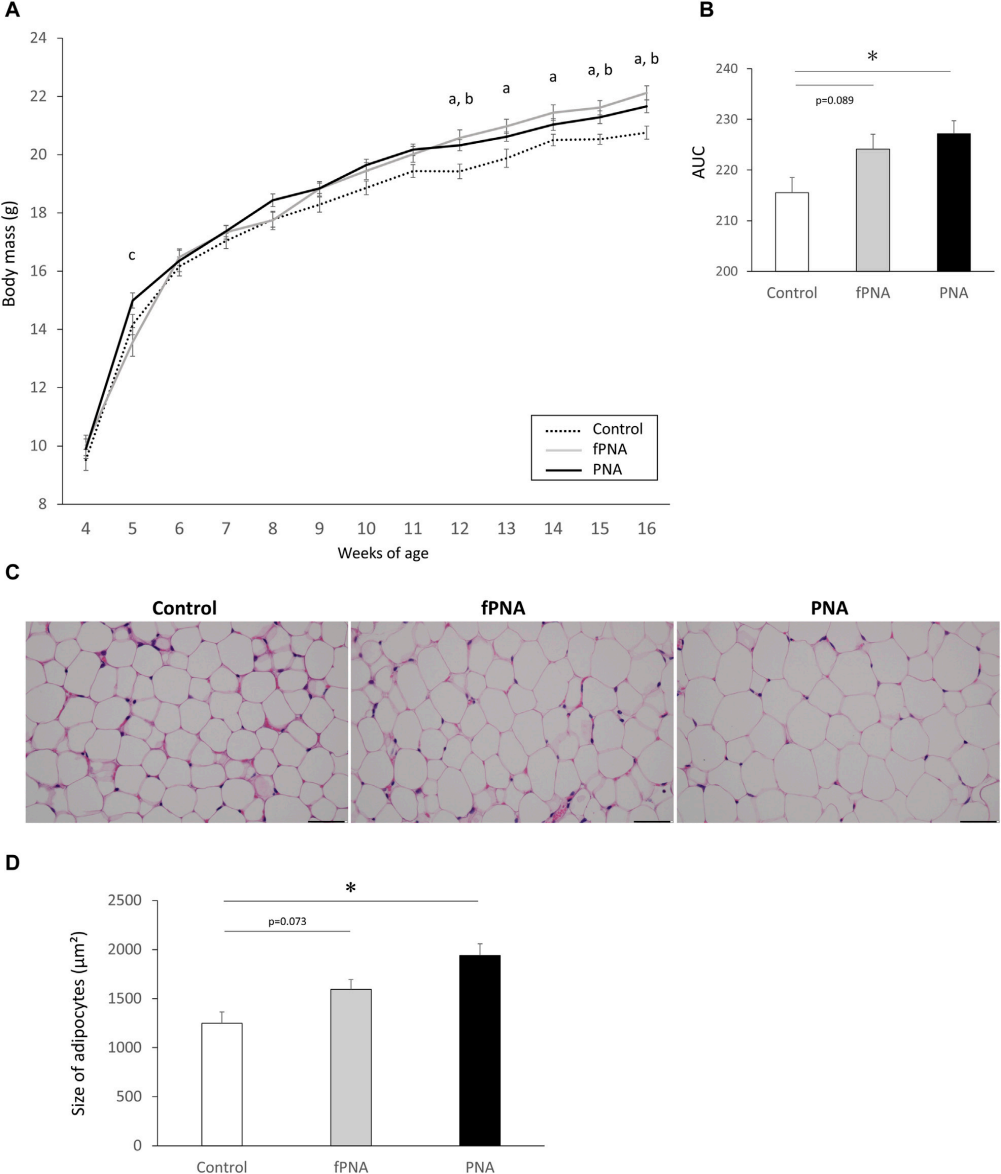
Body mass and the size of parametrial adipocytes. **(A)** Body masses of control, fostered PNA, and PNA offspring between 4 and 16 weeks of age. There were no differences of the body masses of the mice in the three groups at 4 weeks of age (prepuberty); however, fostered PNA and PNA offspring were heavier after 12 weeks (young adulthood) than control offspring. **(B)** Area under the body mass curve (AUC), which was significantly larger in PNA offspring than in control offspring. Fostered PNA offspring showed a similar trend to PNA offspring, but the difference between fostered PNA and control offspring was not significant. **(C)** Representative sections of parametrial adipose, ×40 magnification. **(D)** The size of parametrial adipocytes in the three groups. PNA offspring showed hypertrophy of their parametrial adipocytes at 16 weeks of age and fostered PNA offspring showed a similar trend, but the difference between the fostered PNA and control offspring was not significant. Scale bars: 50 μm. Values are means ± SEMs. Differences in the letters “a,” “b,” and “c” indicate differences at *p* < 0.05 for the fPNA versus the control group, the PNA versus the control group, and the fPNA versus the PNA group, respectively. *p < 0.05 according to one-way-ANOVA, followed by *post-hoc* Tukey-Kramer honest significant difference testing. fPNA, fostered PNA.

At 4 weeks of age (weaning), there were no differences in the body masses of the control, fostered PNA, and PNA groups; however, both the fostered PNA and PNA offspring were heavier than controls from 12 weeks (young adulthood) onwards (Figure 4A). The area under the curve (AUC) for the gain of body mass was significantly larger for PNA offspring than for control offspring (*p* = 0.020), and the fostered PNA offspring showed a similar trend, but the difference between the fostered PNA and control offspring did not reach significance (*p* = 0.089) (Figure 4B). In addition, the parametrial adipocytes of the PNA offspring were significantly larger than those of the controls at 16 weeks (*p* = 0.002), and the fostered PNA offspring showed a similar trend, but again, the difference between the fostered PNA and control offspring did not reach significance (*p* = 0.073) (Figures 4C, D).

### 3.4 Female offspring prenatally exposed to androgen are insulin resistant from 12 weeks of age, regardless of their postnatal early-life environment

**FIGURE 5:**
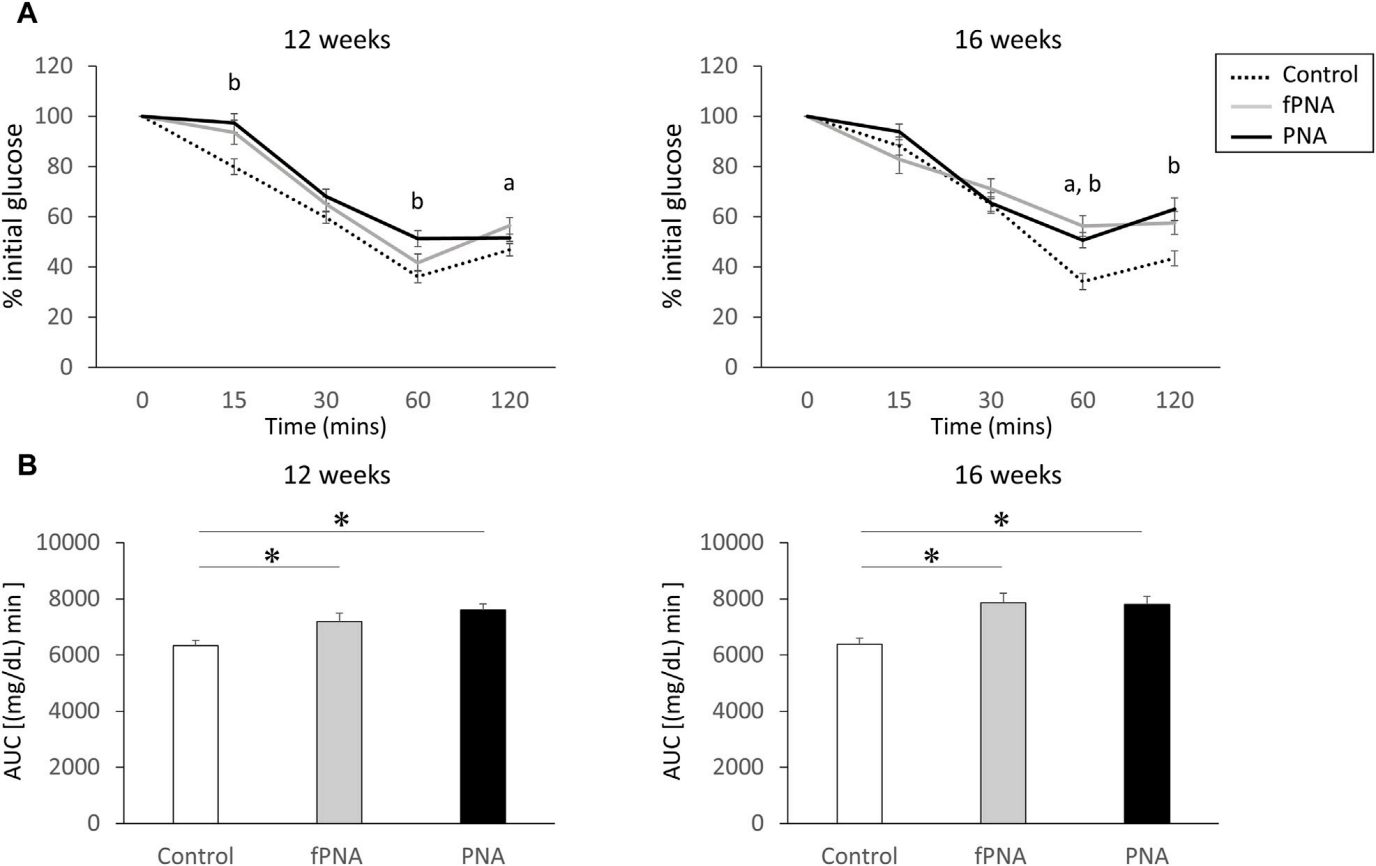
Insulin resistance. **(A)** Results of insulin tolerance testing (ITT) in control, fostered PNA, and PNA offspring at 12 and 16 weeks of age. The insulin-induced reduction in serum glucose concentration was less marked 60 and 120 min after insulin injection in fostered PNA offspring at 12 and 16 weeks of age; and 15, 60, and 120 min after insulin injection in PNA offspring at 12 and 16 weeks of age. **(B)** Area under the serum glucose curve (AUC) during ITT. The AUC was significantly larger in the fostered PNA and PNA offspring than in the control offspring at 12 and 16 weeks of age. This implies lower insulin sensitivity in fostered PNA and PNA offspring from 12 weeks onwards. Values are means ± SEMs. Differences in the letters “a” and “b” indicate *p* < 0.05 for the fPNA *versus* the control group and the PNA *versus* the control group, respectively. *p < 0.05 according to one-way-ANOVA, followed by *post-hoc* Tukey-Kramer honest significant difference testing. fPNA, fostered PNA.

Figure 5A shows the results of insulin tolerance testing (ITT) of the offspring mice. The insulin-induced reduction in serum glucose concentration was less marked at several time points in the fostered PNA and PNA offspring at the ages of 12 and 16 weeks than in control mice. In addition, the AUC for serum glucose during ITT was significantly larger in both the fostered PNA and PNA offspring than in controls at 12 (*p* = 0.022 and *p* = 0.002, respectively) and 16 weeks of age (*p* = 0.027 and *p* = 0.003, respectively) (Figure 5B). This implies that both fostered PNA and PNA offspring have lower insulin sensitivity than controls.

### 3.5 Prenatal androgen exposure alters the alpha-diversity of the gut microbiota of mice between 4 and 8 weeks of age, but this effect is abrogated by changing their postnatal early-life environment

**FIGURE 6:**
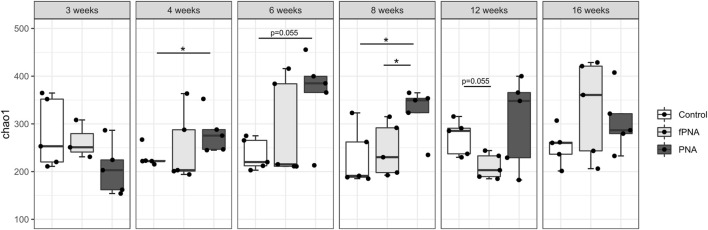
Alpha-diversity of the gut microbiome. The Chao1 index is shown for the control, fostered PNA, and PNA groups. The α-diversities of the groups were compared using the Mann-Whitney U test. The α-diversity of the PNA group was significantly higher than that of control offspring at 4 and 8 weeks of age, and there was a similar trend at 6 weeks of age. However, there were no differences between the fostered PNA and control offspring at all of the time points. *p < 0.05. fPNA, fostered PNA.

The α-diversity of the microbiota, which reflects the richness of the microbial species, was assessed using the Chao1 index. The α-diversity of the PNA group was significantly higher than that of the control offspring at 4 and 8 weeks of age (*p* = 0.032 and *p* = 0.032, respectively), and tended to be higher at 6 weeks of age (*p* = 0.055). There was no significant difference in the α-diversity of the fostered PNA and control offspring at all life stages, but it tended to be lower in fostered PNA mice than in controls at 12 weeks of age (*p* = 0.055) (Figure 6).

### 3.6 Prenatal androgen exposure induces a change in the beta-diversity of the gut microbiota from the early stage of life, but the microbial community is shifted toward that of control offspring by changing the postnatal early-life environment

**FIGURE 7:**
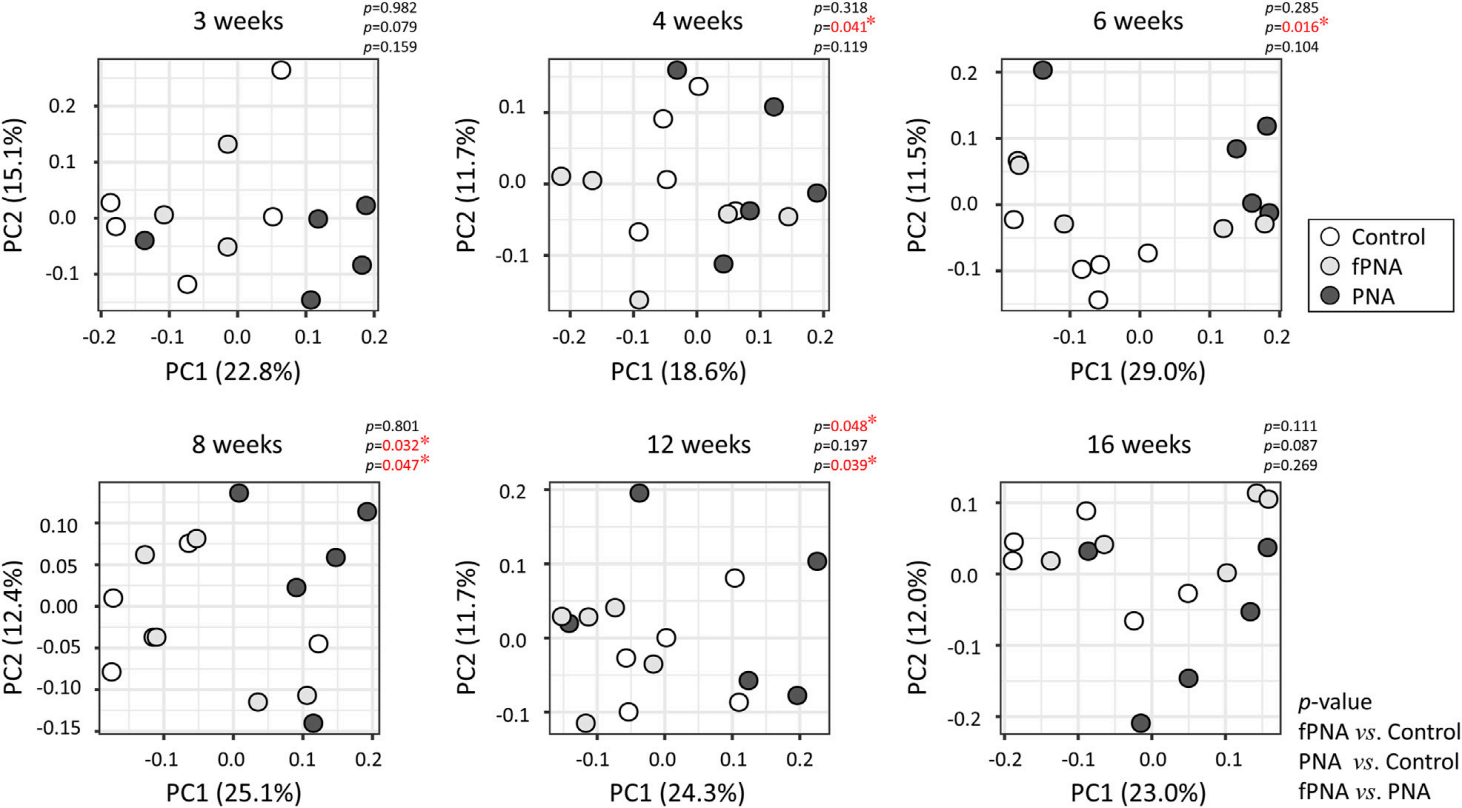
Beta-diversity of the gut microbiome. The β-diversity, reflecting the similarity between groups, was analyzed at each time point using a principal coordinate analysis (PCoA) plot, based on the unweighted UniFrac metric. Each dot represents the composition of the bacterial community in an individual sample. The proportions of the variance that could be explained using principal coordinate (PC) axes 1 and 2 are shown as figures. Significant differences in β-diversity were identified using permutational multivariate analysis of variance (PERMANOVA). The microbial community of the PNA offspring could be differentiated from that of the control offspring at 4, 6, and 8 weeks of age, and a similar trend were found at 3 and 16 weeks of age. By contrast, the microbial community of the fostered PNA offspring could be differentiated from that of control offspring only at 12 weeks of age. Furthermore, the PCoA plots showed significant separation of the fostered PNA group from the PNA group at 8 and 12 weeks of age. *p < 0.05. fPNA, fostered PNA.

The β-diversity, which reflects the similarity between groups, was assessed using the unweighted UniFrac distance and visualized using principal coordinates analysis (PCoA). The PCoA plots showed a significant separation of the PNA group from the control group at 4, 6, and 8 weeks of age (*p* = 0.041, *p* = 0.016, and *p* = 0.032, respectively) (Figure 7), which implies that the microbial communities of the two groups substantially differed at these time points, and similar trends were identified at 3 and 16 weeks of age (*p* = 0.079 and *p* = 0.087, respectively). However, the microbial community of the fostered PNA offspring differed from that of control offspring only at 12 weeks of age. Furthermore, the PCoA plots showed significant separation of the fostered PNA group from the PNA group at 8 and 12 weeks of age.

### 3.7 Prenatal androgen exposure induces changes in composition of the gut microbiota of the mice at all stages of life, but changing their postnatal early-life environment makes it more similar to that of controls in the early stages of life

**FIGURE 8:**
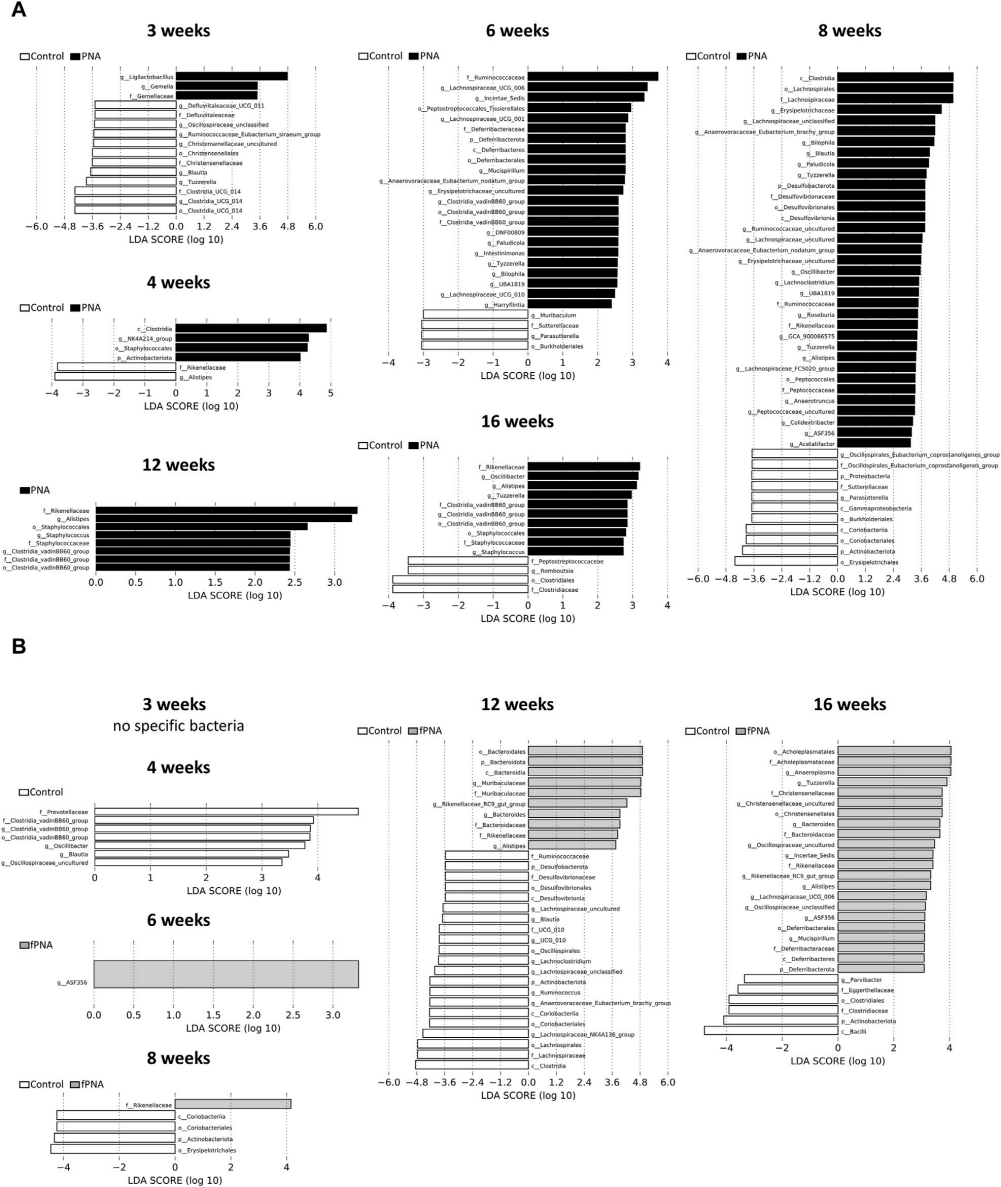
Composition of the gut microbiome. Taxa that were differentially abundant between the **(A)** control and PNA offspring, and **(B)** control and fostered PNA offspring were identified using linear discriminant analysis (LDA) effect size (LEfSe) software. The taxa enriched in the PNA and fostered PNA groups *vs*. the control group are shown in black and gray, respectively. A significance level of < 0.05 and an LDA effect size of >2 were used as the thresholds in LEfSe analysis. Differences in microbial composition were identified in multiple taxa at each time point. Genera that characterized the control and PNA offspring were already apparent from 3 weeks of age and this persisted until 16 weeks of age. However, no or only a few bacterial taxa differed in abundance between the fostered PNA and control offspring between 3 and 8 weeks of ages, whereas many bacteria differed in abundance at 12 and 16 weeks. fPNA, fostered PNA.

The gut microbial compositions of control, fostered PNA, and PNA offspring were analyzed taxonomically at the genus level. Bacterial taxa that significantly differed in abundance in the fostered PNA and PNA offspring *vs*. the control offspring were identified using linear discriminant analysis (LDA) and LDA effect size (LEfSe). The abundances of several bacterial genera significantly differed between the control and PNA groups at all the ages assessed (Figure 8A). On the other hands, only a few bacterial taxa differed between the fostered PNA and control offspring between 4 and 8 weeks of age, and no specific bacteria was detected at 3 weeks of age. However, the abundances of many bacterial taxa differed at 12 and 16 weeks (Figure 8B). There were several bacterial taxa differed consistently between the PNA and fostered PNA offspring across multiple time points: Blautia, Anaerovoracaceae-Eubacterium-nodatum-group, Anaerovoracaceae-Eubacterium-brachy-group, Bilophila, and Erysipelotrichaceae-uncultured were more abundant in the PNA offspring (Supplementary Figure S1), however, all of them, no bacteria were more abundant than control group. Table 1 is a summary of all the bacterial taxa at genus levels that were consistently more or less abundant in the PNA or fostered PNA offspring, compared to controls across multiple time points. Most of them were more abundant in the PNA or fostered PNA offspring after 6 weeks of age: Alistipes were more abundant in both the PNA and fostered PNA offspring. Tuzzerella Anaerovoracaceae-Eubacterium-nodatum-group, Clostridia-vadinBB60-group, Paludicola, Tyzzerella, Bilophila, UBA 1819, Oscillibacter, and *Staphylococcus* were more abundant in PNA group. ASF356, Rikenellaceae-RC9-gut-group, and *Bacteroides* were more abundant in fostered PNA group. On the other hands, Parasutterella was less abundant in PNA offspring at 6 and 8 weeks of age. Blautia was less abundant in the PNA offspring at 3 weeks, and in fostered PNA offspring at 4 and 12 weeks.

**TABLE 1:** Differences in the gut microbial composition of the prenatally androgenized (PNA) and fostered PNA offspring compared to control offspring. All the bacterial taxa that were consistently more or less abundant in the PNA or fostered PNA offspring across multiple time points, identified by linear discriminant analysis (LDA) effect size (LEfSe), are summarized. In LEfSe analysis, a log LDA score >2.0 and *p* < .05 were set as the thresholds. Upward and downward arrows indicate higher and lower abundance in the PNA or fostered PNA group than in the control group, respectively. fPNA, fostered PNA.

Genus level	3 weeks		4 weeks		6 weeks		8 weeks		12 weeks		16 weeks	
	fPNA	PNA	fPNA	PNA	fPNA	PNA	fPNA	PNA	fPNA	PNA	fPNA	PNA
Blautia		**↓**	**↓**					**↑**	**↓**			
Tuzzerella		**↓**						**↑**			**↑**	**↑**
Alistipes				**↓**				**↑**	**↑**	**↑**	**↑**	**↑**
Lachnospiraceae-UCG006						**↑**					**↑**	
Anaerovoracaceae-Eubacterium-nodatum-group						**↑**		**↑**				
Clostridia-vadinBB60-group			**↓**			**↑**				**↑**		**↑**
Paludicola						**↑**		**↑**				
Tyzzerella						**↑**		**↑**				
Bilophila						**↑**		**↑**				
UBA1819						**↑**		**↑**				
Parasutterella						**↓**		**↓**				
Oscillibacter			**↓**					**↑**				**↑**
ASF356					**↑**			**↑**			**↑**	
Staphylococcus										**↑**		**↑**
Rikenellaceae-RC9-gut-group									**↑**		**↑**	
Bacteroides									**↑**		**↑**	

## 4 Discussion

We have shown that the phenotype and gut microbial features of both PNA and fostered PNA female offspring differ from those of control offspring, but the differences are smaller for the fostered PNA offspring. Tables 2, 3 summarize the findings for the PNA and fostered PNA offspring, compared with the control offspring, with respect to PCOS-like phenotypes and the gut microbiota, respectively. These findings indicate that both prenatal androgen exposure itself and the postnatal early-life environment provided by dihydrotestosterone (DHT)-injected mothers contribute to the development of PCOS-like phenotypes and the abnormalities in the gut microbiota that characterize PNA offspring.

**TABLE 2:** Features of polycystic ovary syndrome (PCOS) in prenatally androgenized (PNA) and fostered PNA offspring, compared to control offspring. Upward arrows indicate significantly (*p* < 0.05) higher values than the control group. Arrows with parentheses indicate trends (*p* < 0.10). Horizontal arrows indicate no change was identified compared to the control group. Bold characters for insulin resistance indicate the age when a significant difference (*p* < 0.05) was identified. fPNA, fostered PNA.

		Reproductive phenotypes			Metabolic phenotypes		
		Estrous cyclicity	Atretic follicle	Serum testosterone	Body mass	Adipocytes	Insulin resistance
vs. Control	fPNA	Disrupted	↑	→	(↑)	(↑)	↑12, 16 weeks
	PNA	Disrupted	↑	↑	↑	↑	↑12, 16 weeks

**TABLE 3:** Summary of the differences in the gut microbiome of prenatally androgenized (PNA) and fostered PNA offspring, compared to control offspring. For α-diversity, upward arrows indicate significantly (*p* < 0.05) higher values than the control group. Arrows with parentheses indicate trends (*p* < 0.10). Bold and normal characters indicate the ages when significant differences (*p* < 0.05) and trends (*p* < 0.10), respectively, were identified. For β-diversity, bold characters indicate that the composition of the bacterial communities of the groups (fPNA *vs*. control or PNA *vs*. control) significantly differed at the indicated ages (*p* < 0.05), while normal characters within parentheses indicate trends at the indicated ages (*p* < 0.10). As for the bacterial composition, the number of bacterial genera that were more or less abundant in the fostered PNA or PNA group than in the control group at each time point is shown. Differences in composition were identified using linear discriminant analysis (LDA) effect size (LEfSe), and in LEfSe analysis, a log LDA score >2.0 and *p* < 0.05 were used as the thresholds. fPNA, fostered PNA.

		α-diversity	β-diversity	Composition					
				3w	4w	6w	8w	12w	16w
vs. Control	fPNA	(↑ 12 weeks)	12 weeks	0	4	1	0	12	13
	PNA	↑4, 8 weeks (↑ 6 weeks)	4, 6, 8 weeks (3, 16 weeks)	9	2	17	26	3	6

Fostered PNA offspring had less marked PCOS-like phenotypes than PNA offspring (Table 2). With respect to the reproductive phenotype, PNA offspring exhibited disruption to their estrous cycles from 6 weeks (puberty) onwards, and polycystic ovarian histology and high serum testosterone concentrations at 16 weeks (adulthood); while fostered PNA offspring showed disruption of their estrous cycles from puberty onwards and polycystic ovarian histology in adulthood, but normal serum testosterone concentration in adulthood. With respect to the metabolic phenotype, PNA offspring were heavier and more insulin resistant from 12 weeks of age (young adulthood) onwards, and to show hypertrophy of visceral adipocytes in adulthood. By contrast, the fostered PNA offspring showed insulin resistance in adulthood, but only slightly higher body mass throughout the study period, as shown by analysis of area under the body mass curve, and slightly larger visceral adipocytes in adulthood. The findings in the PNA offspring are consistent with those of previous studies: the reproductive phenotype appears at puberty, whereas the metabolic phenotype appears in young adulthood (Skarra et al., 2017; Kusamoto et al., 2021). The PCOS-like phenotypes in the PNA offspring remained in large part even after changing their postnatal early-life environment by fostering, which suggests that the prenatal environment, rather than the postnatal early-life environment, is critical for the etiology of PCOS in later life.

From a translational point of view, it is noteworthy that fostered PNA offspring had less marked PCOS-like phenotypes than PNA offspring, specifically with respect to serum testosterone concentration and visceral adiposity in adulthood and body mass throughout the study period. The etiology of the reproductive phenotype seems to involve the prenatal environment in contrast to the metabolic phenotype. Of all the reproductive parameters assessed, the reason why only the serum testosterone concentration was improved by changing the postnatal early-life environment is unknown. This may be explained by the characteristics of the PNA model, in which the increase in testosterone concentration is modest (Kusamoto et al., 2021). The present findings suggest that interventions targeting the postnatal early-life environment of offspring exposed to high prenatal androgen concentrations, a feature of the intrauterine environment of mothers with PCOS, may prevent them from developing PCOS phenotypes, and especially the metabolic phenotype, in later life.

PNA offspring exhibited differences in their gut microbiota from that of controls throughout the study period, and these differences were more marked before adolescence than in young adulthood or subsequently, whereas the gut microbiota of the fostered PNA before adolescence was similar to that of controls, and the difference was apparent after young adulthood (Table 3). The gut microbiota of the PNA offspring differed from that of controls with respect to its richness and composition, as demonstrated by analysis of their α- and β-diversity from 4 weeks (prepuberty) to 8 weeks (adolescence) of age. The abnormal composition of the gut microbiota at the genus level in the PNA offspring was apparent before puberty and was present at all the time points thereafter. By contrast, there was no significant difference in the α-diversity of the fostered PNA and control offspring at all the life stages evaluated. The composition of the gut microbiota in the fostered PNA shifted toward that of control offspring from 4 weeks (prepuberty) to 8 weeks (adolescence) of age, as shown by analysis of the β-diversity, while the fostered PNA showed significant differences in their microbial community from both the control and PNA offspring at 12 weeks of age (young adulthood). Similarly, the differences in bacterial composition at the genus level in the fostered PNA *vs*. the control offspring became apparent from young adulthood.

Differences in the richness of the microbial population, the α-diversity, between patients with PCOS and healthy controls have been identified previously. Multiple previous studies of the adult human and rodent gut microbiota have demonstrated lower α-diversity in women with PCOS and rodent models of PCOS than in controls (Lindheim et al., 2017; Liu et al., 2017; Torres et al., 2018; Rizk and Thackray, 2021), and one study that characterized the gut microbiota of adult PNA rats showed higher α-diversity at 25 weeks of age (Sherman et al., 2018). There have been fewer studies of the gut microbiota associated with PCOS in early life, but that of adolescent girls (14–16 years old) with PCOS and obesity showed lower α-diversity than that of girls with obesity but no PCOS (Jobira et al., 2020), and that of female PNA mouse offspring showed higher α-diversity than that of controls during adolescence (Kusamoto et al., 2021). Given that higher α-diversity has been identified in males than in females after puberty (Insenser et al., 2018), the higher α-diversity in PNA offspring of 4, 6, and 8 weeks of age, corresponding to the period between weaning and adolescence, identified in the present study could be attributable to the high testosterone concentration that is present from 6 weeks of age onwards (Kusamoto et al., 2021). Considering that low α-diversity is associated with metabolic abnormalities, such as obesity and type 2 diabetes (Mosca et al., 2016; Menni et al., 2020), it is conceivable that the lack of difference identified in the α-diversity of the PNA offspring and controls from 12 weeks of age in the present study reflects the manifestation of the metabolic phenotype from this time. In contrast to the PNA offspring, the fostered PNA offspring did not show a significant difference in α-diversity from controls prior to adolescence, and it tended to be lower in young adulthood. These differences may be principally attributable to the normalization of the serum testosterone concentration in the fostered PNA offspring.

Differences in the β-diversity of the microbial communities of adult patients with PCOS and healthy controls have also been identified previously (Lindheim et al., 2017; Liu et al., 2017; Torres et al., 2018; Rizk and Thackray, 2021). Although few studies have assessed the β-diversity of patients with PCOS in early life, adolescent girls with PCOS and obesity have also been shown to have microbiota with differing β-diversity to those with obesity but no PCOS (Jobira et al., 2020). In addition, several studies have shown altered gut microbial composition (β-diversity) in rodent models of PCOS in adulthood (Kelley et al., 2016; Moreno-Indias et al., 2016; Sherman et al., 2018; Torres et al., 2019a). In the present study, the differences in the microbial community between the PNA and control groups were marked at 4 (prepuberty), 6 (puberty), and 8 weeks (adolescence) of age, whereas the microbial community of the fostered PNA offspring was similar to that of control offspring at these time points. This finding is consistent with those of previous studies in which rodents with PCOS underwent fecal microbiota transplantation (FMT), which restored a normal gut microbial composition (Guo et al., 2016; Torres et al., 2019b). Interestingly, the microbial composition of the fostered PNA offspring differed from those of both control and PNA offspring at 12 weeks of age (young adulthood). This may be because the normalization of the gut microbial community achieved by fostering during early life does not persist into young adulthood. Alternatively, the effects of the metabolic abnormalities that become apparent in the fostered PNA offspring after young adulthood may override the effects of the normalization of the gut microbial community during early life, even though the metabolic phenotype of these mice is less marked than that of the PNA offspring.

Alterations in the composition of the gut microbiota at the genus level were apparent from as early as 3 weeks of age and persisted throughout the period of study in the PNA female offspring. Various taxa have been reported to be up- or downregulated in adult women with PCOS and in adult rodents with PCOS, depending on the model used (Rizk and Thackray, 2021; Min et al., 2023). In the present study, among the taxa that were found to be different between control, fostered PNA, and PNA offspring, several taxa associated with insulin resistance including Alistipes (Takeuchi et al., 2023), Bilophila (Liu et al., 2021), and Parasutterella (Henneke et al., 2022) were more or less abundant in fostered PNA and PNA offspring compared to control offspring, although whether these bacterial changes were associated with or caused by PCOS-like phenotypes. Although the gut microbiota of humans and rodents differ, alterations in their composition appear relatively early in life in both rodent models and in women who develop PCOS (Kelley et al., 2016; Torres et al., 2019a; Jobira et al., 2020; Kusamoto et al., 2021). It is conceivable that the balance of the entire microbiota and the metabolites produced by the bacteria are more important than the individual bacterial taxa themselves in the development of PCOS, meaning that rodent models and women who develop PCOS have similar characteristics. In contrast to the PNA offspring, the fostered PNA offspring exhibited no or a few differences from control offspring with respect to the abundances of specific bacterial taxa between 3 and 8 weeks of age. The differences in the findings for PNA offspring and fostered PNA offspring indicate that both prenatal androgen exposure and the postnatal nurturing environment before weaning affect the formation of the gut microbiota of prenatally-androgenized individuals before adolescence.

The gut microbial community starts to develop during gestation, and its composition is influenced by various factors such as gestational age, the mode of delivery (vaginal delivery or cesarean section), and the type of feeding (breast milk or artificial milk), as well as maternal conditions during gestation and lactation (Kapourchali and Cresci, 2020). In addition, the prenatal environment, including the mother’s gut microbiota and its metabolites, and the early postnatal environment, may influence the risks of developing certain diseases in the offspring (Kimura et al., 2020). The mechanism by which intrauterine prenatal androgen exposure affects the gut microbiota has yet to be elucidated. In this study, the composition of the gut microbiota of DHT-injected mothers differed from that of control mothers after 3 days of injections (Supplementary Figure S2). The microbial community of the DHT-injected mothers could be differentiated from that of the control mothers. Alternatively, high levels of androgen in the maternal circulation may directly affect the formation of the gut microbiota in the fetus. Many bacterial taxa differed in their abundance between the fostered PNA and control offspring after young adulthood, which may be the result of the development of the metabolic phenotype in the fostered PNA offspring during this period.

It would be intriguing to clarify the contribution of the alterations in the gut microbiota to the development of the PCOS phenotype. In the present study, prenatal androgen exposure resulted in abnormalities in the gut microbiota from prepuberty onwards, and the improvement of the postnatal early-life environment through fostering by a control mother shifted the composition of the gut microbiota toward that of controls before adolescence, while the PCOS-like phenotype induced by prenatal androgen exposure was ameliorated in adulthood. Previous studies that demonstrated the effects of the restoration of a normal gut microbiota on PCOS generated contradictory results: some showed that treatment with healthy FMT or probiotics, or co-housing with normal animals, ameliorated their PCOS-like phenotype, including their estrous cyclicity, serum testosterone concentrations, and body composition (Guo et al., 2016; Torres et al., 2019b), while another study showed that healthy FMT had no effect on the PCOS-like phenotype, despite the composition of the gut microbiota being shifted toward that of controls (Rodriguez Paris et al., 2022). The variability in these findings may be explained by the difference in the models used, the mode of exposure to a healthy gut microbiota, or the timing of the intervention. In the present study, prenatal androgen exposure may have induced abnormalities in the gut microbiota early in its formation and altered its composition prior to adulthood. Fostering by a normal mother, which involves exposure of the pups to their mothers through feces, breast milk, and direct contact, brought the composition of the gut microbiota in the offspring close to that of the control offspring and ameliorated some aspects of the PCOS-like phenotype in later life, but not substantially, which may be explained by the lack of normalization of the gut microbiota. These results indicate that alterations in the initial formation of the gut microbiota that are induced by prenatal androgen exposure, which have long-term effects on its composition, also have implications for the development of PCOS in later life. Further intervention studies targeting the gut microbiota of mothers or their very young female offspring would be helpful to investigate this further.

There were some limitations to the present study. First, the “prenatal environment” in the present study includes both intrauterine prenatal androgen exposure and the exposure of the offspring to their mother’s microbiota through vaginal delivery. Cesarean delivery would be required to separate the effects of these two exposures, but this is difficult in rodents. Second, it should be noted that this study observed the changes in gut microbiota and phenotypes induced by intrauterine androgen exposure in mice and does not fully recapitulate the mechanisms of human PCOS. In addition, the design of the present study was based on the premise that the PNA and fostered PNA offspring were prenatally exposed to androgens. The findings that both the pre- and postnatal environments contribute to the development of PCOS-like phenotypes and the alterations in the gut microbiota in later life cannot be extrapolated to “normal” offspring born to control mothers that are not prenatally exposed to high androgen concentrations.

In conclusion, both prenatal androgen exposure itself and the postnatal early-life environment created by the DHT injection of mothers contribute to the development of PCOS-like phenotypes and the alterations to the gut microbiota in PNA offspring. The PCOS-like phenotypes in later life, and especially the metabolic phenotype that develops after young adulthood, can be ameliorated by interventions during early life. In particular, the composition of the gut microbiota of PNA offspring before adolescence is shifted toward that of controls by interventions in early life. Future studies of the mechanisms by which the alterations in the gut microbiota contribute to the development of PCOS would be helpful for the design of strategies for the prevention of PCOS.

## Data Availability

The datasets presented in this study can be found in online repositories. The names of the repository/repositories and accession number(s) can be found in the article/Supplementary Material.
