# Supplementary material for: Effects of the prenatal and postnatal nurturing environment on the phenotype and gut microbiota of mice with polycystic ovary syndrome induced by prenatal androgen exposure: a cross-fostering study
Source: Front Cell Dev Biol. 2024 Mar 25;12:1365624. doi: 10.3389/fcell.2024.1365624 (PMC10999616; doi:10.3389/fcell.2024.1365624)
Supplement: Supplementary file 1 [file DataSheet1.docx]

Supplementary Material


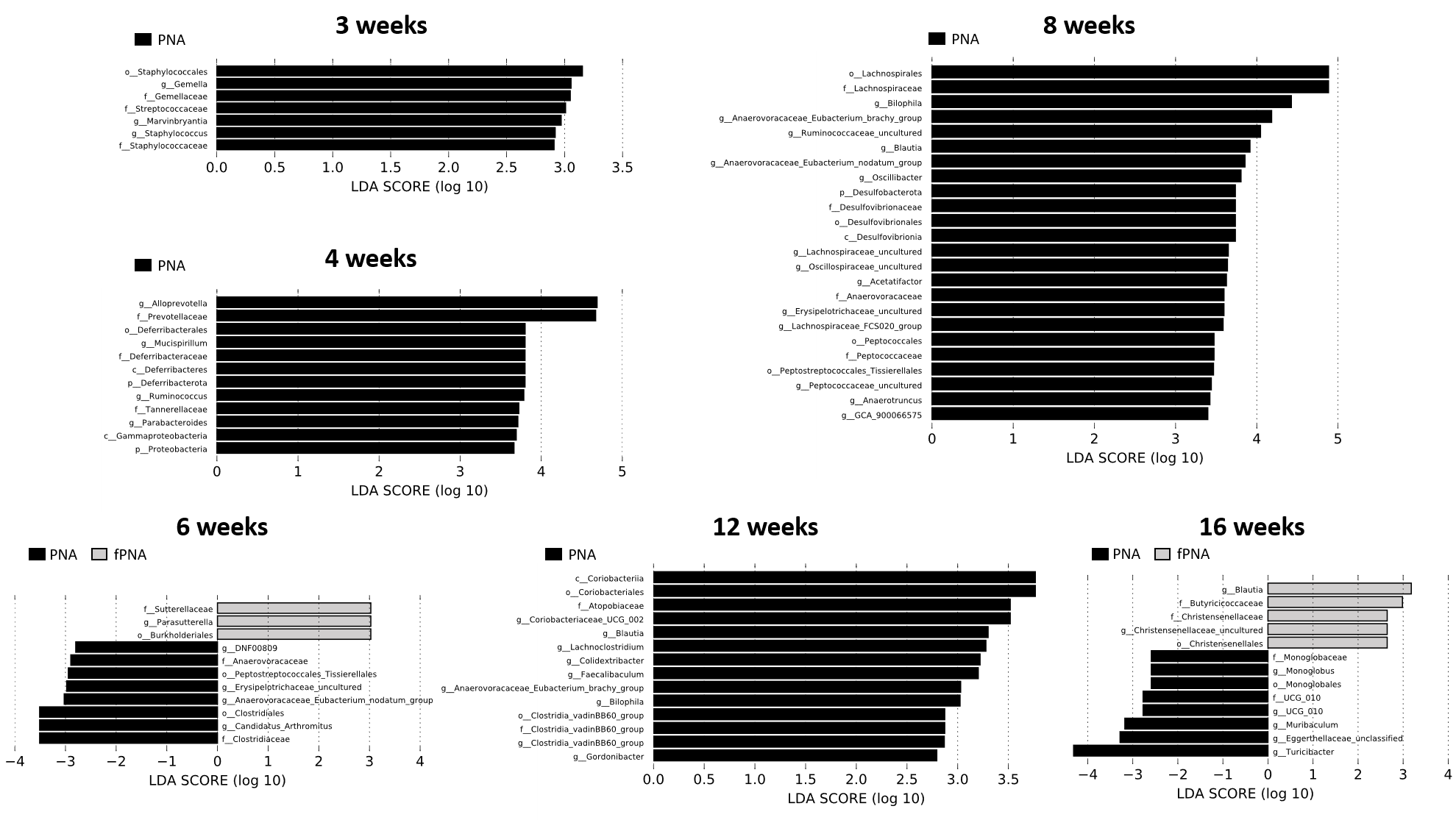


Supplementary Figure 1. The composition of the gut microbiota differed in prenatally androgenized (PNA) and fostered PNA offspring at every time point. Taxa that were differentially abundant between the PNA and fostered PNA offspring were identified using linear discriminant analysis (LDA) effect size (LEfSe) software. Taxa enriched in the PNA and fostered PNA groups are colored by black and gray, respectively. A significance level of < 0.05 and an LDA effect size of >2 were used as the thresholds in LEfSe analysis. Differences in microbial composition were identified in multiple taxa at each time point. fPNA, fostered PNA.


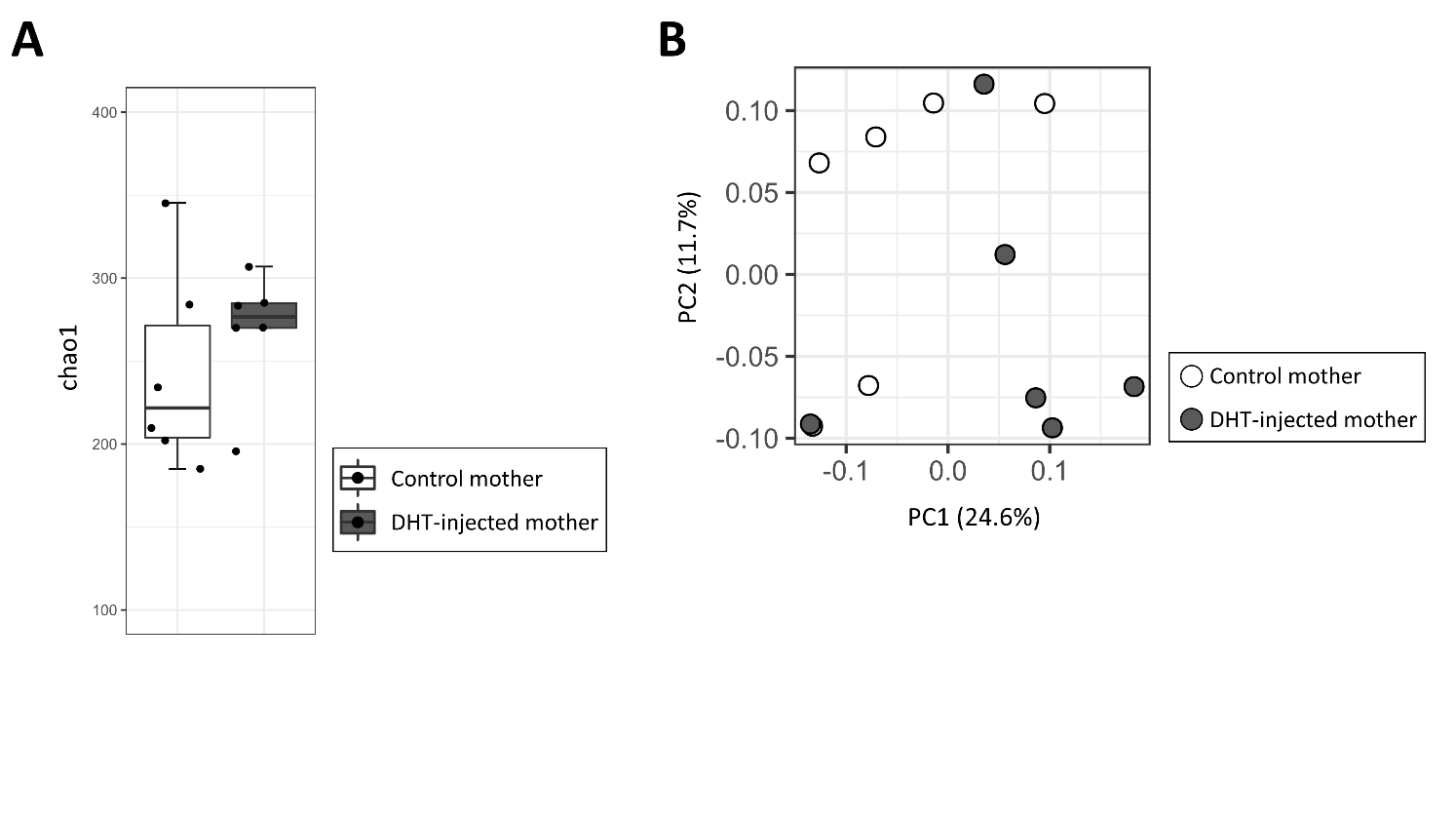


Supplementary Figure 2. Effects of dihydrotestosterone (DHT) injection on the gut microbiota of the mothers. Pregnant dams were subcutaneously (s.c.) injected on days 16, 17, and 18 of gestation with either 0.1 mL sesame oil (control mothers) or 0.1 mL sesame oil containing 250 μg of dihydrotestosterone (DHT-injected mothers). To evaluate the effects of androgen injection on the gut microbiota of the mothers, next generation sequencing and bioinformatic analysis of 16S rRNA genes were performed on DNA samples obtained from the feces of control and DHT-injected mothers on day 19 of gestation. n=5–6/group. (A) Chao1 index for the control and DHT-injected mothers. The α-diversity of the groups was compared using the Mann-Whitney U test. There was no difference between the control and DHT-injected mothers. (B) β-diversity, reflecting the similarity between groups, analyzed using a principal coordinate analysis (PCoA) plot, based on the unweighted UniFrac metric. Each dot represents the composition of the bacterial community in an individual sample. The proportions of the variance that could be explained using principal coordinate (PC) axes 1 and 2 are shown in figures. Differences in β-diversity were identified using permutational multivariate analysis of variance (PERMANOVA). The microbial community of the DHT-injected mothers could be differentiated from that of the control mothers, although the difference was not statistically significant (p=0.073).
